# Antioxidants and Exercise Performance: With a Focus on Vitamin E and C Supplementation

**DOI:** 10.3390/ijerph17228452

**Published:** 2020-11-15

**Authors:** Madalyn Riley Higgins, Azimeh Izadi, Mojtaba Kaviani

**Affiliations:** 1 Faculty of Pure and Applied Science, School of Nutrition and Dietetics, Acadia University, Wolfville, NS B4P 2R6, Canada; 136385h@acadiau.ca; 2 Department of Biochemistry and Diet Therapy, Faculty of Nutrition and Food Sciences, Tabriz University of Medical Sciences, Tabriz 5166/15731, Iran; izadi.sahar7@gmail.com

**Keywords:** sport performance, altitude training, resistance exercise, dietary supplements, free radicals

## Abstract

Antioxidant supplementation, including vitamin E and C supplementation, has recently received recognition among athletes as a possible method for enhancing athletic performance. Increased oxidative stress during exercise results in the production of free radicals, which leads to muscle damage, fatigue, and impaired performance. Despite their negative effects on performance, free radicals may act as signaling molecules enhancing protection against greater physical stress. Current evidence suggests that antioxidant supplementation may impair these adaptations. Apart from athletes training at altitude and those looking for an immediate, short-term performance enhancement, supplementation with vitamin E does not appear to be beneficial. Moreover, the effectiveness of vitamin E and C alone and/or combined on muscle mass and strength have been inconsistent. Given that antioxidant supplements (e.g., vitamin E and C) tend to block anabolic signaling pathways, and thus, impair adaptations to resistance training, special caution should be taken with these supplements. It is recommended that athletes consume a diet rich in fruits and vegetables, which provides vitamins, minerals phytochemicals, and other bioactive compounds to meet the recommended intakes of vitamin E and C.

## 1. Introduction

In addition to rigorous training and diet regimes, many high-level athletes still look for an extra edge to improve their performance, often turning to nutrient supplementation. It has been estimated by the American College of Sports Medicine that approximately 50% of athletes take vitamin supplements with the goal of staying fit and improving endurance [1,2]. Recently, antioxidant supplementation has received attention among athletic populations as a possible method to reduce muscle damage incurred during exercise [3].

Intakes of vitamin C and E vary widely across the world, with the prevalence of individuals with an inadequate intake ranging between 34–95% for vitamin E and 5–65% for vitamin C in different groups, including the general population and athletes [4,5]. The current recommended dietary allowances (RDA) of vitamin E for adults is 15 mg for both males and females. For vitamin C, the RDA for adults aged 19 years or older is 75 mg for females and 90 mg for males. It has been reported that most athlete users of antioxidant supplements already have an adequate intake of vitamin C and E and meet the RDAs for both vitamins [6]. Over the last decade, the use of antioxidant supplementation has been questioned, as they seem to inhibit or attenuate the signaling of important adaptations such as muscle mitochondrial biogenesis and hypertrophy [7]. This paper will review the role of free radicals and antioxidants, as well as their effects on athletic performance with a primary focus on vitamin E alone and in combination with vitamin C. 

## 2. Antioxidants and Sports Performance 

Regular exercise has many demonstrated benefits including reducing the risk of diseases such as type II diabetes, cancer, and dementia, as well as improving the function of our organs, in particular the skeletal muscle [7]. However, during intense exercise, free radical or reactive oxygen and nitrogen species (RONS) production increases and may inhibit muscular contractile function leading to muscle fatigue and performance impairment [8]. Given the role of antioxidants in protection against free radicals, it has become common practice among athletes to consume antioxidant supplements to combat muscle damage and fatigue and to enhance performance [7]. Although antioxidants play an important role in the protection from RONS, evidence suggests that antioxidant supplementation may impair exercise training adaptations. The concern is that the reactive species generated during exercise might be implicated in the improvement in aerobic capacity and muscle hypertrophy, through stimulating molecular pathways via proteins, including peroxisome proliferator-activated receptor-c coactivator (PGC1-α) and mitogen-activated protein kinases (MAPK) [9,10,11]. For example, according to Ristow et al. [12], vitamin E (400 IU/day) and vitamin C (1000 mg/day) consumption prevents the induction of PGC1-α and mitochondrial biogenesis, as well as key endogenous antioxidant enzymes in human skeletal muscle. In this sense, increased mitochondrial biogenesis is a major adaptation to exercise training in skeletal muscle and PGC1-α is considered the master regulator in mitochondrial biogenesis [13].

Conversely, some authors suggested positive effects of a transient increased level of RONS induced by exercise. In fact, the RONS is implicated in regulating muscle contractile activity, in addition, RONS stimulates muscle regeneration [14] and improves vasodilation during exercise [15]. However, high concentrations of RONS and oxidative stress increase inflammation and damage to cells and tissues [16]. 

To sum up, the balance between RONS and antioxidant systems is important. Despite their high training demands, evidence shows that many endurance athletes have diets which contain insufficient antioxidants to support their physical activity demands [17]. Given their low intakes of antioxidants, it is often believed that endurance athletes should take antioxidant supplements. However, given the many demonstrated health benefits of exercise, it appears to be unlikely that free radical production via exercise will negatively impact performance in the long term [17].

### 2.1. Reactive Oxygen and Nitrogen Species 

Reactive oxygen and nitrogen species (RONS), also called free radicals, are produced in the body continually via oxidative metabolism [8]. The terms free radicals and RONS will be used interchangeably throughout this paper. RONS arise when there is insufficient oxygen to complete a reduction resulting in the creation of a free radical [18]. Free radicals are highly reactive due to an unpaired electron in their outer orbital [8]. Due to their reactivity, high concentrations of free radicals can cause damage to lipids, protein, and DNA [8]. During exercise, increased oxygen consumption leads to an increase in RONS production [19]. This increase in RONS production during exercise may contribute to muscle damage, impaired immunity, and fatigue [19]. Muscle damage, including lipid peroxidation, incurred from RONS during acute exercise has been suggested as a possible cause of delayed onset muscle soreness (DOMS) and exercise performance impairment [20,21]. Despite the damage they may cause, free radicals are also essential for proper physiological functioning acting as intracellular messengers [17]. 

Although high levels of RONS demonstrate negative effects on exercise performance, more recently, their role in positive cellular adaptation to stress and athletic training has been investigated [19]. Free radicals may act as signaling molecules for muscle function regulation and adaptation via the upregulation of protective proteins [22]. This upregulation in protective proteins allows for increased protection against future stress and free radical exposure [22]. The extent to which these reactive species are harmful versus beneficial depends on various factors, including exercise duration and intensity, an athlete’s nutritional and training status, and age [19]. Lower doses of RONS appear to be beneficial for training adaptations during acute performance [22]. However, increased RONS production in skeletal muscle is implicated in muscle damage and impaired muscle performance [23]. Regarding the training status, it has recently been shown [24] that sprinter and endurance master athletes have better redox balance and inflammatory status, compared to the age-matched control, but worse than the untrained adults. Regarding the exercise mode, sprinters presented a better antioxidant capacity than both the controls and endurance runners, whereas the nitric-oxide profile (as a marker of endothelial function) was better for endurance runners and lower for the controls. Endurance runners have shown a better nitric-oxide profile, as a marker of endothelial function, whereas sprinters had a better redox balance and cytokines profile. Therefore, a personalized supplementation with respect to the type of exercise and training status seems reasonable given the discrepancies existing in the current literature [25]. 

### 2.2. Exogenous and Endogenous Antioxidants

Antioxidants are compounds which help protect cellular organs from oxidative damage incurred via free radicals [26]. There are many different antioxidants which can be classified as endogenous (produced in the body) or exogenous/dietary (taken in from external sources). Antioxidants can also be classified as enzymatic (catalytically remove free radicals) or non-enzymatic (remove free radicals in ways other than a catalytic reaction) [17]. Antioxidants protect against oxidative stress by converting free radicals into non-radicals thereby reducing their reactivity, or by preventing the conversion of inactive radicals into more damaging species [17].

Endogenous antioxidants are proteins produced by the body and can be either enzymatic or non-enzymatic [17]. Endogenous enzymatic antioxidants include superoxide dismutase (SOD), catalase (CAT), and glutathione peroxide (GPX) [17,22]. The major non-enzymatic endogenous antioxidant is glutathione (GSH) [17]. Endogenous antioxidants production increases following exercise and plays a role in protecting cells from oxidative damage [22]. Well-trained athletes possess higher levels of endogenous antioxidants in their muscles than athletes with less training as a result of training adaptations [17]. Consequently, those who train irregularly or at lower intensities such as recreational athletes, will likely have less protection against oxidative stress [18].

Most fruits and vegetables contain a variety of exogenous antioxidants. However, humans can also acquire exogenous antioxidants though other food sources such as nuts and seeds [17]. Important exogenous antioxidants which play a role in protection from free radicals include vitamin E, vitamin C, vitamin A, polyphenols, and some minerals (Zinc, Manganese, Cupper, Selenium) [22]. Exogenous antioxidants are obtained in the diet through foods such as fruits and vegetables or through dietary supplements [17]. 

The oxidative capacity of antioxidants varies based on the type of free radicals [8]. Exogenous antioxidants, such as vitamin E and C, are non-targeted free radical scavenging antioxidants, whereas our endogenous antioxidants are more complex and allow for a more controlled, localized effect [7]. Endogenous antioxidants are our first line of defense against free radicals, while exogenous antioxidants including vitamin E and C, act as a second line of defense offering further protection [27]. Recently, supporting endogenous antioxidants through oral ingestion of exogenous antioxidants has received attention as a possible strategy to reduce oxidative stress and decrease muscle damage sustained during acute performance [26]. Oxidative stress and muscle damage are thought to impair performance and recovery, making the promise of antioxidant supplementation to reduce this effect, of interest to athletes. 

### 2.3. Antioxidants and Impaired Adaptations to Training 

Although muscle damage and fatigue are undesirable to athletes and prolonged oxidative stress may lead to cellular damage, RONS role as signaling molecules may result in favorable adaptations to exercise training [7]. The physiological stress, including exposure to RONS, which occurs during acute exercise results in skeletal muscle adaptations enabling muscles to cope with further stressors in the future [7]. One such training adaptation that may be impaired is the body’s enhanced endogenous antioxidant production. In response to endurance exercise training, endogenous antioxidants production is increased and may provide sufficient protection without the need to increase exogenous antioxidant intake [17]. 

## 3. Search Strategy

A systematic literature search was performed up to October 2020 in PubMed, Scopus, and Web of Sciences based on the following keywords: Vitamin C, ascorbic acid, vitamin E, tocopherol, exercise, training, exercise performance, aerobic, resistance training, endurance, strength, muscle hypertrophy, and adaptation. Inclusion criteria were vitamin C or vitamin E supplementation (alone or together) combined with an exercise training program (both acute and long-term supplementation and training program). Two investigators (M.R.H. and A.I.) independently screened the abstracts and titles and retrieved the relevant full texts to assess eligibility based on the inclusion criteria. The full-text articles included were also searched manually for any additional studies. 

## 4. Vitamin E as An Antioxidant Supplement 

There are many different types of exogenous antioxidants athletes may take, although vitamin E appears to be one of the most widely available [19]. Vitamin E refers to lipid soluble compounds including four tocopherols and four tocotrienols, with α-tocopherol being the most biologically available and most well-known form [22]. Tocopherols and tocotrienols act as potent free-radical scavengers in membranes and lipoproteins, they quench fatty acid peroxyl radicals and yield tocopheroxyl radicals, the resulting tocopheroxyl radicals may be reduced by an appropriate reducing agent such as ubiquinol or vitamin C to regenerate vitamin E. Although many antioxidants are found in nature, vitamin E is one of the most widely distributed [17]. Vitamin E is found in lipid rich structures such as the sarcoplasmic reticulum, where it scavenges free radicals produced by the mitochondria, thereby reducing lipid peroxidation and membrane damage [8]. 

### 4.1. Food Sources Versus Supplements 

Although exogenous antioxidants can be found in lipid based membranes as well as in aqueous phase, no endogenous antioxidants exist within lipid based cell membranes, therefore, making it essential to acquire some antioxidants from the diet [17]. To ensure a variety of exogenous antioxidants, it is recommended that athletes consume a diet rich in fruits and vegetables [17]. Along with their high antioxidant content, fruits and vegetables may also provide benefits for athletes given that many of the additional bioactive compounds they contain are not found in single dose pharmacological antioxidant supplements [12,28]. It has also been suggested that the different kinds of antioxidants found in plant foods may act synergistically allowing them to have more positive effects than single, mega dose antioxidant supplements [27]. 

Given that vitamin E is a fat soluble vitamin, athletes following lower fat diets may have an impaired vitamin E intake as well as absorption [29]. Sacheck et al. [30] investigated the dietary intake of vitamin E among collegiate female rowers following a low fat versus a high fat diet and found that those in the low fat group consumed significantly less vitamin E (2.9 mg vitamin E/day) than those in the high fat group (9.8 mg vitamin E/day). In addition to a reduced intake of vitamin E on a lower fat diet, athletes in the study likely would have an impaired absorption of vitamin E as well. An impaired absorption of vitamin E as a result of following a low fat diet may result in insufficient levels of vitamin E, meaning that some athletes may benefit from additional vitamin E through supplementation [29]. 

Koivisto et al. [28] investigated whether high antioxidant intakes from food affect the adaptive response to athletic training, as well as whether increasing the antioxidant intake via antioxidant rich foods would affect adaptive responses in elite athletes following altitude training. Daily antioxidant rich foods consumed in the study included 50 g of dried berries and fruits, a 750 mL fruit, vegetable, and berry smoothie, 40 g walnuts, and 40 g dark chocolate (>70% cocoa content) Compared to a placebo group, no differences were reported in VO_2_ max, erythropoietin, or hemoglobin mass following an antioxidant rich diet. The authors concluded that enhancing the antioxidant concentration via increased consumption of antioxidant rich foods does not impair adaptive responses to training, thereby contracting results from studies on antioxidant supplementation. This further supports the idea explaining how antioxidants from foods rather than supplements may help athletes receive adaptation benefits from oxidative stress while keeping oxidation low enough to avoid harm. More recently, Koivisto et al. [31] reported that consumption of antioxidant-rich foods increased antioxidant capacity and decreased some of the altitude-induced inflammatory biomarkers in elite athletes. Koivisto et al. [31] found that consumption of antioxidant-rich foods had no effect on the oxidative stress or acute cytokine responses to exercise stress-tests at altitude.

It is reported [32] that a docosahexaenoic acid (DHA) and vitamin E-enriched beverage consumed at 1 L per day, 5 days/week, for 5 weeks containing 45.7 ± 27.7 mg/L alpha-tocopherol, did not alter the performance parameters such as blood lactate and fatigue during a maximal exercise test. The enriched beverage which was provided to both young and senior athletes, protected plasma lipid oxidative damage, although it enhanced nitrative damage in erythrocytes in the young athletes after exercise. The gene expression of peripheral blood mononuclear cells (PBCM) antioxidant enzymes was enhanced after acute exercise only among the young athletes supplemented with the beverage. Despite beverage supplementation demonstrating a reduction in the plasma oxidative damage and an enhanced adaptive PBMC antioxidant response in young athletes, no effect was seen among the senior athletes. In summary, the effects of functional beverage supplementation were age-dependent and require more studies. In another study by Capó et al. [33], performance (measured as exercise time) was not affected by enriched beverage supplementation. More recently, Hoene et al. [34] suggested a cautious use of vitamin E as a dietary supplement, since they observed that a vitamin E-enriched diet interferes with the adaptation process to exercise in mice. However, Górnicka et al. [35] suggested that an impaired α-tocopherol status and its adequate intake is required to preserve an optimal status to prevent the skeletal and cardiac muscles, as well as the testes from damage, since in their study, α-tocopherol reduced lipid peroxidation in mice subjected to physical effort. Yi et al. [36] investigated the effects of 75 g of almonds (a good source of vitamin E) consumed as single pre-exercise supplements over 4 weeks, and observed the improved performance (measured as distance travelled). Similarly, acute almond supplementation (60 g, 2 h before exercise) is reported to enhance performance in endurance exercise in the trained subjects [37]. An animal study also [38] reported that tocotrienol-rich fraction (TRF) increased liver and muscle glycogen and reduced the exercise-induced oxidative stress, as well as blood lactate forced on swimming rats.

Mega doses of vitamin E via supplementation can result in large increases in body stores of the vitamin [17]. Receiving too much vitamin E through food alone is nearly impossible, however, a state of vitamin E toxicity can be met through supplementation resulting in gastric distress and an increased risk of bleeding due to the role of vitamin E as an anticoagulant [17]. Despite the risk of toxicity, athletes who do not consume a varied and balanced diet may benefit from antioxidant supplementation to meet the recommended dietary allowances (RDAs) of antioxidant vitamins including vitamin E [39]. In addition, if reducing oxidative stress and inflammation have priority, adapting a balanced diet with additional mixed fruit, vegetables, and berries, as well as supplementing with antioxidant-enriched beverages is indicated.

### 4.2. Supplementation with Vitamin E Alone and Combined with Vitamin C and Exercise Performance

Vitamin E supplementation, often combined with vitamin C, is common among athletes given their combined antioxidant effect [40]. Vitamin E is a fat-soluble vitamin which includes four tocopherols and four tocotrienols with α-tocopherol in the most biologically available and well-studied form [22]. Vitamin E is a powerful antioxidant which is capable of donating hydrogen atoms to free radicals including superoxide and hydroxyl radicals, converting them to a more stable form, and preventing lipid peroxidation and membrane damage [8]. Similarly, vitamin C, a hydro soluble vitamin, protects against free radical production by scavenging free radicals [8]. Vitamin E and C work in conjunction with each other, with vitamin C helping to recycle vitamin E back to a reduced state and enabling it to continue to oxidize free radicals [8].

Under most dietary conditions, vitamin E concentrations in the body are relatively low and with low vitamin E stores shown to increase muscular fatigue; increasing vitamin E concentrations through supplementation is a promising practice for athletes [17]. In a review of 10 studies investigating the effects of vitamin E and/or C supplementation on chronic exercise and exercise adaptation, Nikolaidis et al. (2012) [26] noted mixed results. Of the studies reviewed on antioxidant supplementation, two of them reported an ergolytic effect, six showed no effect, and a further two reported an ergogenic effect [7]. Of note, two of the studies reporting a positive effect used rodent models and cannot be directly applied to humans or exercise performance [26]. One older study by Akova et al. (2001) [41] tested the effects of vitamin E supplementation on muscular performance among sedentary females noting no effects following supplementation. Zoppi et al. (2006) [42] also reported no effect on antioxidant enzymes concentrations or performance following supplementation with vitamin E and C on elite soccer players. According to Silva et al. [43], vitamin E supplementation could provide protection from inflammation, exercise-induced muscular and oxidative damage, fatigue, and muscle force loss induced by exercise. 

Current evidence on the effects of vitamin E supplementation on endurance outcomes is equivocal. Rodent studies [44,45] indicated hindering effects of vitamin E supplementation on exercise-induced mitochondrial biogenesis and antioxidant enzymes in skeletal muscle. Several human studies reported no effect on exercise performance outcomes following supplementation with vitamin C and/or E during endurance exercise training [9,46,47,48]. However, there are some human studies that have shown negative effects of combined vitamin C and E on the adaptive responses of skeletal muscle to endurance training, such as attenuated mRNA responses in mitochondrial proteins and antioxidant enzymes [9,40]. To sum up, there is convincing evidence that vitamin C and E, taken alone or in combination blunts some skeletal muscle adaptations to endurance training. There is no evidence that vitamin C and/or vitamin E supplementation has negative effects on maximum oxygen uptake (VO_2max_) as a measure of performance and training adaptations, though. In their 2014 study, Paulson et al. [40] reported no effect on VO_2_maxfollowing supplementation with vitamin E and C despite impaired cellular adaptations. Paulson et al. [40] also found that following an endurance training protocol, those in the placebo group showed increased fat oxidation and reduced heart rate while performing submaximal exercise, whereas those supplementing with vitamin C and E showed no improvements in fat oxidation or heart rate. In another study on the effects of vitamin E and C supplementation on endurance performance, Merry and Ristow (2016) [7] noted similar findings reporting no effect of supplementation on VO_2_max. A recent systematic review concluded that vitamin C and/or vitamin E has no negative effect on VO_2_ max [49].

More recently, there has been some investigation on the effects of antioxidant supplementation on muscle hypertrophy. The current evidence suggests that supplementation with vitamin E and C does not affect hypertrophy in young participants and athletes [19]. However, supplementation of vitamin C may attenuate lean mass gains in older adults [7]. Bjørnsen et al. [50] observed less increase in total mass gain following vitamin C (500 mg) and vitamin E (117.5 mg) supplementation compared with the placebo group. On the contrary, Bobeuf et al. [51] investigated the effects of co-administration of vitamin C (1000 mg) and E (600 mg) combined with strength training for 6 months in sedentary healthy elderly participants. Authors observed that only participants who combined strength training with supplementation gained fat-free mass (+1.5 kg) by the end of study. Authors concluded that vitamin C and E supplementation might have reduced damage and/or increased protein synthesis induced by muscle contraction associated with strength training. However, they did not measure the oxidation or synthesis of protein. Bobeuf et al. subsequently [52] reported that 6 months of resistance exercise (3 times a week) in healthy elderly participants had no significant effect on lean mass, while the combination of resistance exercise with antioxidant supplementation (600 mg vitamin E and 1000 mg vitamin C per day) significantly increased lean mass. The study by Bobeuf et al. likely has more power due to the larger sample size. In this sense, a short-term high-dose vitamin C and E supplementation (vitamin C: 2000 mg/day, vitamin E: 1400 IU/day; 4 days) has been effective to attenuate exercise-induced muscle damage and inflammatory response during and after competitive Olympic Taekwondo (TKD) matches in elite athletes [53]. However, Cumming et al. [54] reported that vitamin C and E supplementation did not affect acute stress responses or long-term training adaptations in the heat shock proteins or endogenous antioxidants among trained adults. 

Recently, it is suggested that redox processes might contribute to resistance training adaptations and muscle hypertrophy [25]. It is worth nothing that although Paulsen et al. [10] showed that in healthy young adults, subjected to a heavy-load resistance training, vitamin C and E supplementation did not impair lean body mass gain, or the acute changes in protein synthesis in muscle, the increased phosphorylation of extracellular signal-regulated kinase 1/2 (ERK1/2) and ribosomal protein S6 kinase (p70S6k), induced by training was blunted. It should be noted that P70S6k and ERK 1/2 are involved in anabolic cellular transduction pathways leading to muscle hypertrophy [25]. Dutra et al. [55] investigated the effects of strength training combined with antioxidant supplementation on muscle performance and thickness among young females. The authors demonstrated that, although vitamin E in combination with vitamin C did not affect quadriceps muscle thickness, performance measurements (i.e., peak torque and total work) were negatively affected by supplementation. The authors concluded that excess vitamin C and E may reduce the phosphorylation of important hypertrophy pathways mediated by RONS, such as p38, ERK1/2, and p70S6K, which support this explanation. On the other hand, the study by Bobeuf et al. [52] was carried out in aged populations and reported a beneficial effect of antioxidant supplementation. Therefore, it is hypothesized that, under pro-oxidative conditions (ageing), exogenous antioxidants restore redox balance [25] and provide health benefits. 

With regard to the importance of achieving and maintaining optimal body weight in many sports, the necessity for weight loss is a very common situation among athletes [56]. However, this is important to note that attempting to lose weight/fat might be associated with reduced dietary fat intake, which in turn is associated with a decreased alpha-tocopherol status [57]. Of note, according to some cohort studies, there is a positive association between the plasma α:γ-tocopherol ratio and fat-free mass percentage (FFM%) and BMI [58], and the dietary vitamin E intake is associated with greater fat-free mass and (FFM)% mass [59]. 

Regarding the effects of vitamin E alone or combined with vitamin C supplementation (in conjunction with strength training) on strength gains, five studies [50,52,55,60,61] have been done and reported neither positive nor negative effects on strength gain. A recent comprehensive meta-analysis [49] provided evidence that vitamin E supplementation alone or combined with vitamin C neither enhances nor blunts exercise-induced training adaptations, including changes in aerobic capacity, muscle strength, or lean mass and endurance performance. However, it is unclear whether in the state of deficiency or inadequate intake, these supplements would be beneficial for this purpose. 

Although few studies have been conducted on elite athletes, Gillam et al. [62] investigated whether there is a threshold for the serum and membrane vitamin E level to maintain the integrity of cell membranes following a bout of intense aerobic exercise. Their study demonstrated that vitamin E levels are lower in elite male runners compared to untrained individuals. Therefore, to prevent perturbations in the membrane integrity induced by training, the levels of serum and membrane α-tocopherol should be higher than 12 and 3 mg/L, respectively, while the reference range for plasma α-tocopherol level is 8.1–13.0 mg/L. Gillam et al. [62] concluded that supplementation with vitamin E may assist the recovery in elite athletes.

Many studies investigating the effects of vitamin E supplementation, on both athletes and nonathletes, also include vitamin C in their supplementation protocol. Vitamin C and E are key components in an interacting network of the antioxidant defense system [63]. Similar to the function of vitamin E as an antioxidant, vitamin C has the ability to protect against lipid peroxidation by scavenging free radicals [63].

**Figure 1:**
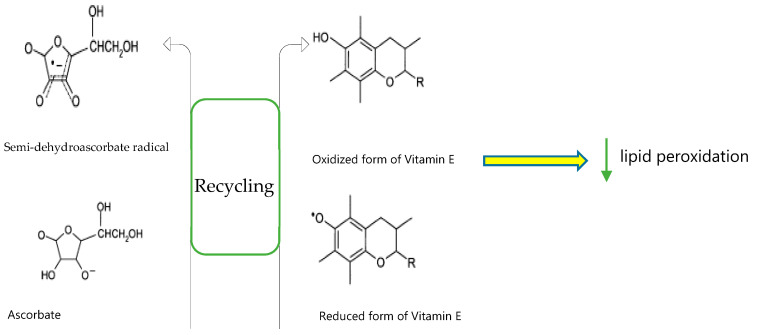
Vitamin C acting as a reducing agent to recycle vitamin E to protect against lipid peroxidation.

The interaction between both vitamins E and C is based on the ‘vitamin E recycling’. With vitamin E recycling, the vitamin E, tocopherol, reacts with a peroxyl radical to form a tocopheryl radical, which in turn is regenerated by vitamin C (Figure 1) [8]. This vitamin E recycling requires a supply of vitamin C and is often why these nutrients will be consumed simultaneously via supplementation [63]. More recently, Jungert et al. [64] investigated the determinants and interrelation between plasma concentrations of vitamin C and E in the elderly. For plasma vitamin C concentrations, the use of supplements, physical activity, fat-free mass, and plasma α-tocopherol were the main determinants. Age, the use of supplements, the use of lipid-modifying drugs, and plasma vitamin C were the main determinants for the α-tocopherol/total cholesterol ratio [64]. The results emphasize the idea of an interrelation between plasma levels of vitamin C and E, and also suggest an association between physical activity and fat-free mass with vitamin C and E status. 

As vitamin E and C work closely together, and this interrelation has been shown previously [65,66], therefore, both vitamin C and E will also be explored throughout this paper. 

In a study by Morrison et al. [9], healthy young men were randomly allocated to take a placebo or antioxidant (vitamin C (2 × 500 mg/day) and E (400 IU/day)) for 4 weeks. Following acute exercise, vitamin C and E supplementation did not decrease skeletal muscle oxidative stress or increase gene expression of mitochondrial biogenesis markers. However, supplementation with vitamin C and E mitigated skeletal muscle adaptations indicated by the superoxide dismutase (SOD) activity and mitochondrial transcription factor A (TFAM). Studies investigating the effects of vitamin E with or without vitamin C on exercise performance outcomes in humans and animals are summarized in Table 1 and Table 2, respectively.

### 4.3. Acute Versus Chronic Supplementation with Vitamin E

Many factors including the type of antioxidant, duration of supplementation, and type of training determine the effect of antioxidant supplementation on exercise performance [7]. The duration of antioxidant supplementation may vary greatly among athletes with some choosing to only the supplement acutely during periods of intense exercise, while others may continually supplement throughout their training phases. 

Acute or single dose antioxidant supplementation during high intensity, short recovery intervals, has been shown to improve performance by reducing oxidative stress and speeding up recovery [7]. Merry and Ristow (2016) [7] suggested that antioxidant supplementation may only benefit athletic performance acutely and when an immediate performance enhancement is desired, and adaptation is less important such as during a championship game or performance. Supporting the argument that only acute supplementation appears beneficial, Bentley et al. [8] stated that following a chronic antioxidant supplementation regimen, training adaptations and future exercise performance may be impaired. 

An animal study also reported that acute vitamin E supplementation enhanced the endurance of exercise-induced vasodilation in response to acetylcholine [29]. However, chronic vitamin E supplementation had no further effects on vascular function compared to exercise training alone [81]. A possible exception where chronic supplementation, or supplementation which occurs more than once in succession, may provide additional benefits is a tournament style situation where several bouts of high-intensity exercise are endured within a short period of time. To explain the differences between acute and chronic antioxidant supplementation, Bentley et al. explained that likely a dose dependent relationship exists for antioxidant supplements suggesting that an optimal amount of antioxidants depends on the type and duration of exercise undertaken. 

In a review on chronic vitamin E consumption among athletes, Braakhuis and Hopkins [19] reported a trend towards performance impairment rather than enhancement. In contrast, Roberts et al. [81] reported performance enhancement following antioxidant supplementation and stated that high doses (1600 IU) of vitamin E for 16 weeks was the minimum dose required to demonstrate beneficial effects. Although this study also did not investigate the effects of vitamin E on athletes, Roberts et al. [82] suggested that after supplementing vitamin E at 1600 IU each day for 6 weeks, a reduction in oxidative stress can be seen, which reduces the risk of diseases associated with high levels of oxidative stress. As being physically active has many demonstrated health benefits and no physical activity was administered as part of this study, care should be taken before applying these results to athletic populations.

Redox-signaling pathways are involved in both acute and chronic responses of skeletal muscle to exercise, including muscle insulin sensitivity and glucose uptake [83], mitochondrial biogenesis [40,44], muscle contraction force [10,84], and muscle hypertrophy [50]. In addition, both acute and chronic exercise modulate endogenous antioxidant enzyme levels, therefore, enhancing the capacity of skeletal muscle to neutralize RONS [85]. Moreover, the common antioxidant supplementation may also improve the capacity to decrease deleterious effects of increased RONS generation during exercise [84]. Benefits might relate to an ameliorating effect of antioxidant supplementation on exercise-induced muscle damage and delayed onset of muscle soreness (DOMS) [71]. RONS are also implicated in premature muscular fatigue during sustained submaximal muscle contraction and exercise [86]. According to Mason et al., antioxidant supplementation might help delay muscular fatigue and improve exercise performance [84].

Although there are potential benefits of antioxidant supplementation in exercising humans, according to some evidence, supplementation with vitamin C and E might impair rather than improve some acute and chronic adaptive responses to exercise [9,10,40]. In particular, antioxidant supplementation has been found in some studies to impair some adaptive responses to resistance [10,50] and endurance exercise training [9,40]. 

## 5. Supplementation with Vitamin C and Exercise Performance

The effects of vitamin C on muscle strength and function have been investigated in several studies, however, results so far have been inconsistent. For example, Gomez-Cabrera et al. [11] reported that vitamin C supplementation decreased endurance capacity (running time) and suppressed the exercise-induced increase in mitochondrial biogenesis (PGC-1α). Furthermore, in the human experiment, vitamin C supplementation suppressed the exercise-induced in VO_2_max. However, according to Evans et al. [87], vitamin C supplementation is capable of increasing a peak muscular pushing force (PMF) in untrained individuals.

In our review, we concluded that muscle strength and function are not influenced by vitamin C supplementation. However, individuals with a poor vitamin C status appear to benefit most from supplementation [88], as vitamin C has already been reported to be beneficial in increasing exercise performance in individuals with low baseline levels of vitamin C [89]. Paschalis et al. [89] investigated whether baseline concentrations of vitamin C determine the efficacy of supplementation in enhancing physical performance. A summary of studies investigating the effects of vitamin C on exercise performance outcomes and oxidative stress markers is presented in Table 3. 

## 6. Effects Vitamin E and C Supplementation: Environment and Physiological Factors 

### 6.1. Altitude Training 

Altitude training increases RONS production and oxidative stress making antioxidant supplementation of interest to athletes training at altitude [28]. Within the past decade, there has been a dramatic increase in antioxidant supplementation among athletes particularly among endurance athletes training at altitude [28]. The primary characteristic of high altitude is decreased oxygen availability, which can impair physical and mental performance among those living and/or training at high altitudes [18]. To account for the decreased oxygen availability, our body increases important oxygen carrying components of our blood including hemoglobin and hematocrit [18]. As a result of its role in the maintenance of red blood cell (RBC) structure, vitamin E may provide performance enhancing benefits to athletes training at altitude [19]. Braakhuis and Hopkins (2015) [19] suggested that when training at altitude, RBC lysis may occur which may be prevented by vitamin E supplementation. In their review of studies investigating the effects of vitamin E supplementation on athletic performance, Takanami et al. (2000) [29] noted that at sea level, vitamin E appears to be of little ergogenic aid to athletic individuals, however, at altitude it may benefit physical performance. As the altitude increases, ultraviolet (UV) radiation, both UV-A and UV-B, are simultaneously increased [18]. This increase in UV-B radiation has been postulated as a major reason for increased oxidative stress, particularly lipid peroxidation, at high altitudes [18]. Vitamin E levels are increased in those whose skin receives chronic sun exposure suggesting that vitamin E may help protect against the oxidative damage, which occurs via UV-B radiation at an increased rate at high elevations [18]. 

Although nutrient deficiencies may be of concern for athletes even at low altitudes, at high altitudes they are most certainly of concern given the increased stress incurred on the body [18]. In an investigation of two different high altitude mountaineer expeditions, Simon-Schnass [18] found that nutrient intake, including vitamin E, was below the recommended intakes. Although these studies were not focused on high performance athletes, the results suggest that there is a higher risk of insufficient nutrient intake at high altitudes as a result of many factors including price and weight of food, availability of fresh produce, as well as reduced appetite [18]. Due to low intakes, and increased oxidative stress at altitude, Simon-Schnass [18] stated that it may be advisable for athletes to supplement with vitamin E when training at an increased elevation.

Additionally, early studies [95,96] provided evidence on the beneficial effects of vitamin E on performance at altitude. However, Subudhi et al. [97] reported that supplementation with vitamin E (400 IU per day), had no significant effect on markers of oxidative stress induced by increased energy expenditure at high altitudes. An animal study [77] investigated the effects of vitamin E and C in protecting against lung damage caused by acute swimming at different altitudes. While acute forced swimming significantly increased thiobarbituric acid reactive substances (TBARS) levels and resulted in a significant decrease in activities of SOD, and CAT, combined vitamin E and C supplementation was effective to ameliorate exhaustive swimming and high altitude-associated lung injury. More recently, Santos et al. [72] investigated the effects of vitamin E (250 mg) supplementation on muscle damage and inflammation after moderate exercise in hypoxia simulating an altitude of 4200 m, and reported that supplementation with vitamin E at 1 h before exercise decreases cell damage markers after exercise in hypoxia and reduces the concentration of inflammatory cytokines, suggesting a potential protective effect against inflammation caused by exercise at altitude.

A recent animal study [79] investigated the effects of vitamin E on the immune changes caused by oxidative stress in hypobaric hypoxia (HH) at high altitudes. The results indicate that vitamin E, by its reactive oxygen species quenching effects, blocks the immune changes mediated by free-radicals at high altitudes in a dose-dependent manner. In this regard, vitamin E is the most important lipid-soluble antioxidant that assists in the membranes integrity maintenance and has a direct effect on the functions of immune cells [98]. Moreover, it should be reminded that the reduction in oxygen availability in the mitochondrial electron transport chain in a hypobaric hypoxia state, such as a high altitude, increases the production of free radicals, which are damaging to the cell membrane [99,100].

### 6.2. Mitochondrial Biogenesis and Antioxidant Induction

With regards to the antioxidant effects of vitamin C and E in humans, Yfanti et al. have reported that supplementation with vitamin C (500 mg/day) and vitamin E (400 IU/day) does not interfere with the training-induced adaptations, such as increased mitochondrial proteins and antioxidant enzymes [48,60,69,70]. In contrast, several studies have observed the blunting effects of combined vitamin C and E skeletal muscle adaptations to endurance training [9,12,40]. Ristow et al. [12] have shown that the combined vitamin C (1 g/day) and vitamin E (400 IU/day) supplementation decreased mRNA expression in antioxidant enzymes and several biomarkers of mitochondrial biogenesis following endurance training. Paulsen et al. [40] reported that supplementation with 1 g/day vitamin C and 260 IU/day vitamin E attenuated the increase in COX IV protein abundance and the cytosolic (but not whole cell) levels of PGC-1α in response to training. It is also reported that the combined vitamin C (1 g/day) and vitamin E (400 IU/day) supplementation attenuates the increase in skeletal muscle mitochondrial TFAM protein abundance and SOD enzyme activity [9]. However, the increased citrate synthase activity indicates mitochondrial content levels [101], and its involvement in skeletal muscle adaptations was not attenuated. Thus, collectively, there is now evidence that 1 g/day of vitamin C in combination with vitamin E (400 IU/day) in humans hinders some, but not all of the skeletal muscle adaptations to exercise. However, much of this hampering effect has been attributed to the higher dose of 1 g/day of vitamin C, rather than any effect of vitamin E [84]. Therefore, further studies are needed to investigate if this blunting of some training adaptations is also observed with 1 g/day of vitamin C alone. 

More importantly, despite the potential impairment of some cellular adaptations involved in mitochondrial biogenesis and antioxidant defenses, there are no data available that the combined 1 g/day of vitamin C and 400 IU/day vitamin E supplementation have a negative effect on VO_2_ max or endurance performance. Wyckelsma et al. [73] investigated effects of a 4-week supplementation of vitamin E (235 mg/day) in combination with vitamin C (1 g/day) on several markers of training adaptations and exercise performance in elderly adults following a 3-week sprint interval training on a cycle ergometer. The results showed blunted changes in mRNA expression of ROS-related, inflammatory, and mitochondria proteins in the vitamin groups versus placebo while no significant differences were found in VO_2max_ and maximal power out. From mechanistical point of view, authors suggested that some of the RONS-dependent gene and protein expressions of sarcoplasmic Ca2+ handling proteins which play key roles in mitochondrial biogenesis signaling pathways were blunted by the vitamin supplementation. It was also noted that no performance enhancement in both groups might be attributed to the training period (i.e., 3 weeks), age, and fitness status of participants. 

It is noteworthy to mentioned that there are several factors including RONS concentration, duration of exposure, and training status of individuals determining to what extent RONS exerts positive or negative effects. Gene polymorphisms are another important factor determining the impact of elevated RONS on muscle damage [102]. Approximately 165 autosomal genes, five on the × chromosome, and 17 mitochondrial genes have been identified to contribute to exercise performance. Given the presence of polymorphisms within antioxidant genes which is linked to cellular damage and may result in muscle damage, further studies are needed in this area [103]. 

### 6.3. Skeletal Muscle Contraction Force

An animal study reported that co-administration of vitamin E and α-lipoic acid impaired muscle contractile force in unfatigued, but not fatigued skeletal muscle. Further experiments revealed that the impairment in force production was mostly mediated by vitamin E [74]. It is suggested [85] that since in an unfatigued muscle the redox state is more reduced following antioxidant supplementation, submaximal force production would have been impaired. 

Combined vitamin C (500 mg/day) and vitamin E (1200 IU/day) supplementation enhanced the rate of recovery of the maximal knee extensor voluntary isometric contraction force following intense eccentric knee extension exercises [67]. However, another study reported that co-supplementation with vitamin C (1 g/day) and vitamin E (260 IU/day) did not improve the maximal voluntary knee extensor force recovery after an acute exercise bout [10]. The combination of vitamin C and E supplementation impaired maximal strength development during resistance training in the biceps muscle group and mitigated exercise-induced activation of ERK1/2, MAP kinases p38 MAPK, and p70S6k in the skeletal muscle [10]. Other recent studies investigating the effects of combined vitamin C and E supplementation and resistance training over 3–6 months, have reported no impairment in strength performance following supplementation [52,61]. Overall, the effects of vitamin C and E supplementation on the skeletal muscle contractile function and force production are inconsistent and require future studies. It should be noted that some studies have evaluated the effects of vitamin E on the recovery of muscle contraction force following exercise. Overall, the available evidence on the effects of vitamin E alone or combined with vitamin C against exercise-induced muscle damage are not conclusive [3].

### 6.4. Skeletal Muscle Hypertrophy

Bjørnsen et al. [50] reported that combined vitamin C (500 mg/day) and vitamin E (175 IU/day) supplementation during 12 weeks of resistance training attenuated the gains in leg lean mass and rectus femoris thickness. As well as total lean mass, no significant between-group difference was observed for other body segment masses and muscle thicknesses after training [50]. In contrast, another study [52] reported a significant gain in lean mass only when resistance training was combined with vitamin C (1 g/day) and vitamin E (400 IU/day) supplementation. However, it should be noted that the aforementioned studies were conducted among the elderly and the interpretation of findings related to RONS/antioxidants in this population may not be representative of an athletic population, and therefore, drawing a conclusion for athletes is difficult. Paulsen et al. [10] observed that supplementation with vitamin C (1 g/day) and vitamin E (350 IU/day) during 10 weeks of resistance exercise training had no significant improving effect on lean body mass accretion or muscle group cross-sectional areas in young adults. Although the fractional protein synthetic rate was also unaltered, the acute exercise-induced activation of p70S6k 842 and MAP kinases p38 MAPK and ERK1/2 was attenuated. Interestingly, the antioxidant supplementation attenuated protein degradation as indicated by the attenuation of the post-exercise increase in activation of the ubiquitin proteasome pathway. This latter finding needs future investigation. The contradictory evidence on the effects of vitamin E alone or combined with vitamin C on the overload-induced activation of kinases involved in protein synthesis and protein degradation implies that redox-related signaling pathways in human skeletal muscle hypertrophy remains to be clearly investigated in future studies. More studies are needed to investigate the effects of vitamin E on muscle hypertrophy and strength performance. 

## 7. Conclusions

Drawing clear conclusions on the effects of antioxidant supplementation, including vitamin E with or without vitamin C, is difficult due to variations in the fitness status of the participants, supplementation protocol (type, dosage, duration, timing), type of exercise used, and gender [22]. With very few studies investigating the effects of vitamin E on female participants, more research is needed on the effects of vitamin E on female athletes before any conclusions can be made [26]. As vitamin E supplementation is often combined with additional antioxidants, namely vitamin C, conclusions on the effects of vitamin E alone cannot be made and additional research is warranted. Growing evidence suggests that antioxidant supplementation may impair muscle mitochondrial biogenesis and muscle hypertrophy [7]. Chronic supplementation with vitamin E has been shown to impair athletic performance and is not currently recommended for athletes [19]. Current research fails to show any consistent, positive effect of vitamin E supplementation on health or athletic performance for most athletes [104]. 

Vitamin E supplementation does show promise in two areas of athletic performance. The first area which vitamin E supplementation shows potential benefits, is for athletes participating in altitude training. Supplementation with vitamin E has demonstrated positive effects on athletes training at altitude via a reduction in RBC deformation, however, results still remain inconclusive and further research is warranted [19]. The second area of interest is acute supplementation. Acute antioxidant supplementation has been shown to improve performance during high intensity exercise with short recovery intervals [7]. Supplementation with antioxidants has been suggested to benefit athletes when performance adaptations are not the main focus and immediate enhanced performance is desired. Although acute antioxidant supplementation research appears positive, little research has focused exclusively on acute vitamin E supplementation. 

Based on current evidence, with the possible exception of athletes partaking in altitude training or acute, high stakes performances, vitamin E supplementation, with or without vitamin C, may not provide additional benefits for athletes. To ensure antioxidants needs are met, athletes should instead focus on consuming a diet high in fruits, vegetables, and other plant foods which are rich in antioxidants, as well as other potentially beneficial compounds not found in high dose antioxidant supplements. 

Overall, the effects of vitamin C and E on muscle mass and strength have been inconsistent. As antioxidant supplements (e.g., vitamin E and C) tend to block anabolic signaling pathways, and thus, impair adaptations to resistance training, special caution should be taken with these supplements. Nonetheless, the effects of antioxidants on muscle mass/strength might also depend on the oxidative stress/antioxidants balance of the subject [105].Moreover, among the mechanisms involved in regulating the redox balance, some polymorphisms in genes of antioxidants are associated with cellular damage [102]. While determining the role of the oxidative state and the effects of antioxidants in different types of exercise such as resistance or endurance training, as well as in different categories of athletes such as recreationally active or elite remains to be fully elucidated. Thus, a personalized supplementation approach would be highly recommended. 

## Figures and Tables

**Table 1:** Effects of vitamin E supplementation, with or without vitamin C, on exercise performance.

Study	Participants	Exercise	Timing	Supplementation	Result
Morrison et al. 2015 [9]	Healthy, young men (n = 11)	Ten, 4 min intervals at 90% VO_2_ peak interspersed by 2 min active rest at 50 W	Once per day for 4 weeks	Vitamin C (2 × 500 mg/day) and vitamin E (400 IU/day) (placebo added)	VO_2_ peak and *W* max were significantly increased with no effect of supplementation. Rate of perceived exertion was reduced independent of supplementation
Paulsen et al. 2014 [10]	Recreationally strength trained men (n = 21) and women (n = 11)	4 × 10 RM leg press and knee extension with 1 min rest between sets and 3 min rest between exercises	1–3 h before training and 1 h following training	Vitamin C (1000 mg/day) and vitamin E (235 mg/day) (placebo added)	Supplementation did not blunt muscle hypertrophy, but measurements of muscle strength were lower following supplementation
Ristow et al. 2009 [12]	Previously untrained (n = 19) and pretrained (n = 20) healthy, young males	Four-week physical exercise intervention	Once per day for 4 weeks	Vitamin C (1000 mg/day) and vitamin E (400 IU/day) (placebo added)	Exercise increased parameters of insulin sensitivity in both groups only in absence of antioxidants
de Oliveira et al. 2019 [21]	Football athletes (n = 21)	A protocol consisting of plyometric jumping and strength resistance sets to exhaustion	After 7 days of supplementation, athletes were submitted to training protocol	vitamin C (500 mg/d) and E (400 UI/d) for 15 days	Although antioxidant supplementation reduced oxidative stress, it did not attenuate elevated markers of muscle damage or muscle soreness do not exert any ergogenic effect on football performance
Paulsen et al. 2014 [40]	Recreationally endurance trained (n = 45) and untrained (n = 14) men and women	VO_2_ max treadmill test at 5.3% elevation, and 20 shuttle run beep test	1–3 h before every training session and 1 h after on training days, and in the morning and evening on non-training days	Ascorbic acid (1000 mg/day) and DL α-tocopherol acetate (235 mg/day) (placebo added)	VO_2_ max and shuttle test performance was increased in both groups
Akova et al. 2001 [41]	Sedentary, healthy women (n = 18)	Submaximal cycling (50%) followed by maximal concentric-eccentric combined contractions to measure maximal work force of the dominant knee	Once per day across the duration of two menstrual cycles	Alpha-tocopherol (300 mg/day) (placebo added)	No effect of vitamin E on muscle performance
Zoppi et al. 2006 [42]	Young male soccer players (n = 5)	Lactate maximum speed protocol, one rep max two-legged knee extension, and a 30 m maximal sprint test	Four equal doses every day for 90 days	Ascorbic acid (1000 mg/day) and α-tocopherol (800 mg/day) (placebo added)	No effect on aerobic capacity, strength, or speed
Yfanti et al. 2010 [48]	Healthy, physically active males (n = 21)	Incremental exercise test on a cadence-independent cycle ergometer for VO_2_ max and Pmax	Once per day at breakfast for 4 weeks prior to training then for 12 weeks during cycling training	Vitamin C (500 mg/day) and vitamin E (400 IU/day) (placebo added)	No effect on P_max_ or VO_2_ max
Bjørnsen et al. 2016 [50]	Elderly men (n = 34)	One repetition max (1 RM) leg extension	500 mg vitamin C and 117.5 mg vitamin E before and after training 3 times per week for 3 weeks	Vitamin C (1000 mg/day) and vitamin E (235 mg/day) (placebo added)	Supplementation blunted muscular some adaptations (lean mass gains)
Bobeuf et al. 2011 [52]	Sedentary older men (n = 27) and women (n = 30)	Resistance training 3 days per week for 6 months	Daily for 6 months	Vitamin C (1000 mg/day) and vitamin E (400 IU/day) (placebo added)	No effect on strength gains
Chou et al. 2018 [53]	Elite male taekwondo (TKD) athletes (n = 18)	Four TKD matches against weight matched competitors	Twice daily 3 days before and the day of competition	Vitamin C (2000 mg/day) and vitamin E (1400 IU/day) (placebo added)	Supplementation attenuated circulating creatine kinase and myoglobin. Antioxidant supplementation suppressed exercise provided RBC hemolysis and systemic inflammation
Cumming et al. 2017 [54]	Physically active male (n = 18) and female (n = 10)	A 4x10 RM leg press and knee extension with 1 min rest between sets and 3 min rest between exercises	Two pills 1–3 h before training and 2 pills in the first hour after training	Vitamin C (1000 mg/day) and vitamin E (235 mg/day) (placebo added)	No effect on acute stress responses
Yfanti et al. 2017 [60]	Healthy men (n = 16)	Isometric knee extensor peak at 90° knee flexion	Once per day at breakfast for 5 weeks prior to training and for 4 weeks during eccentric exercise training	Vitamin C (1 g/day) and vitamin E (400 IU/day) (placebo added)	Peak torque increased in both groups with no significant difference between groups
Theodorou et al. 2011 [61]	Recreationally trained healthy men (n = 28)	Five sets of 15 eccentric maximal contractions with each leg on an isokinetic dynamometer	Once daily for 11 weeks at breakfast	Vitamin C (1 g/day) and vitamin E (400 IU/day) (placebo added)	No significant supplement and time effect on muscle function, redox status, or hemolysis observed
Shafat et al. 2004 [67]	Moderately active males (n = 12)	Thirty sets of 10 eccentric knee extensions	Once per day for 37 days	Vitamin C (500 mg/day) and vitamin E (1200 IU/day) (placebo added)	Supplementation reduced the deficit in muscle function experienced during and after bouts of eccentric muscle contractions
Nalbant et al. 2009 [68]	Older adults (n = 57)	three sessions of walking exercise per week	Six months	Vitamin E (900 IU/day)	Exercise alone or combined with vitamin E supplementation improved 6-min walk, chair stand, arm curl tests.
Yfanti et al. 2010 [69]	Moderately trained young men (n = 21)	VO_2_ max cadence dependent cycle test	Daily supplementation for 16 weeks	Vitamin C (500 mg/day) and vitamin E (400 IU/day) (placebo added)	No effect on VO_2_ max or maximum power output
Yfanti et al. 2012 [70]	Healthy, physically active males (n = 21)	One hour at 65% max power on a cycle ergometer	Once per day at breakfast for 16 weeks	Vitamin C (500 mg/day) and vitamin E (400 IU/day) (placebo added)	Supplementation does not further decrease interlukin-6 levels following endurance training
He et al. 2015 [71]	Moderately trained males (n = 22)	40 min downhill run at 65–70% VO_2_ max	Daily for 2 weeks	Vitamin C (1000 mg/day) and vitamin E (400 IU/day)	Supplementation enhanced repeated bout effect as evidenced by attenuation of biomarkers of muscle damage and greater antioxidant capacity
Santos et al. 2018 [72]	Healthy, physically active males (n = 9)	VO_2_ max treadmill test to exhaustion under normal conditions and hypoxia conditions	One hour before exercise	Vitamin E (250 mg) (placebo added)	A reduction was seen in creatine kinase (CK) and lactate dehydrogenase levels.
Wyckelsma et al. 2020 [73]	Recreationally active elderly (mean age 65, n = 18)	nine sessions (three sessions/week for 3 weeks)	On the training days, tablets were taken at least 1 h before the training session	vitamin C (1 g daily) and vitamin E (235 mg daily), treatments were initiated 7 days before the first sprint interval training (SIT) session	Supplementation with antioxidants vitamin C and E blunts SIT-induced cellular signaling in skeletal muscle of elderly individuals

**Table 2:** Effects of vitamin E supplementation, with or without vitamin C, on exercise performance and oxidative stress markers in animals.

Study	Participants	Exercise	Supplementation	Result
Hoene et al. 2018 [34]	Male mice	One hour of treadmill running	Diet supplemented with 100 mg/kg vitamin C and 2000 IU/kg vitamin E (as _-tocopheryl acetate) for 4 weeks	The increase in circulatory free fatty acids 1-h post exercise was blunted vitamin E. Upregulation of several exercise-responsive transcripts was attenuated by vitamin E.
Górnicka et al. 2019 [35]	Male Wistar rats	Fifteen minutes of treadmill running/day	Two mg/day of vitamin E as α-tocopherol acetate for 14 days	Vitamin E supplementation significantly reduced thiobarbituric acid reactive substance (TBARS) in the muscles and heart.
Lee et al. 2009 [38]	Male Wistar rats	Forced swimming 10 h after the last treatment	25 or 50 mg/kg of tocotrienol-rich fraction (TRF), or 25 mg/kg D-α-tocopherol (T-25) for 28 days	TRF improved endurance capacity indicated by longer duration of swimming and reduce the exercise-induced oxidative stress.
Strobel et al. 2011 [44]	Male Wistar rats	Treadmill running 4 days/week for 14 weeks	Vitamin E (1000 mg/kg) and α-lipoic acid (1.6 g/kg) fortified feed	No effect on the changes of exercise induced markers of mitochondrial biogenesis and mitochondrial proteins
Venditti et al. 2014 [45]	Male Wistar rats	Swimming 5 days/week for 10 weeks	Vitamin E (700 mg/kg) fortified food	Vitamin E supplementation attenuated training induced declines in mitochondrial respiration.
Coombes et al. 2001 [74]	Antiox rats	A fatigue protocol (30 min)	10,000 IU vitamin E/kg diet and 1.65 g/kg and α-lipoic acid for 8 weeks	A decline in skeletal muscle force production at low stimulation frequencies
Ryan et al. 2010 [75]	Young and aged Male rats	Three times weekly for 4.5 weeks using 80 maximal stretch–shortening contractions per session	Diet supplemented with Vitamin E (DL-alpha tocopheryl acetate; 30,000 mg/kg) and Vitamin C (Lascorbic acid; 2% by weight)	Supplementing with vitamin E and C reduced oxidative damage markers (e.g., malondialdehyde) associated with aging.
Kyparos et al. 2011 [76]	Male Wistar rats	90 min of intermittent downhill running on a motor-driven treadmill	Vitamin E was administered by daily intraperitoneal injections of 100 mg/kg body mass of DL-α-tocopheryl acetate for 5 consecutive days prior to exercise	Vitamin E supplementation resulted in a higher soleus muscle single-twitch tension immediately post-exercise compared to the placebo condition. No effect of vitamin E supplementation on eccentric exercise-induced muscle damage.
Al-Hashem 2012 [77]	Male rats	Acute forced exhaustive swimming stress for a duration of 2.5 h in glass tanks (in both low and high altitude)	A single dose of 25 mg/kg of vitamin E and 20 mg/kg of vitamin C 1 h before the experimental procedure	Co-ingestion of vitamin E and C resulted in lower activities of SOD and catalase in the lungs under high and low altitude conditions. measured at both altitudes
Picklo et al. 2015 [78]	Male obese rats	Running 5days/week for 12 weeks on a motorized wheel	Dietary supplementation with vitamin E (0.4 g α-tocopherol acetate/kg) and vitamin C (0.5 g/kg) during a high fat diet	No difference in insulin area under curve was found between the conditions. Exercise combined with vitamin C and vitamin E resulted in a higher mitochondrial DNA content versus exercise alone or high fat diet alone conditions.
Goswami and Ghosh. 2019 [79]	Male Albino rats	The rats were exposed to simulated conditions of hypobaric hypoxia (HH) for 8 h daily for 6 consecutive days	Two equal daily doses of vitamin E (first half of the total dose, i.e., 10, 20, and 30 mg/kg of vitamin E) was given orally 5–6 min before the exposure to HH and the second half was given immediately after	Vitamin E in a dose-dependent manner blocked some of the immune changes such as, phagocytic activity of white blood cell, cytotoxic activity of splenic mononuclear cell. Also, corticosterone levels was reduced by vitamin E.
Fagan et al. 2020 [80]	horses	Six-week conditioning program	Horses divided to three groups and fed the control diet plus (1) 1000 IU/day synthetic α [44] tocopherol, (2) 4000 IU/day synthetic α-tocopherol, or (3) 4000 IU/day RRR-α-tocopherol (natural source)	Vitamin E improved some oxidative and inflammatory responses.

**Table 3:** Effects of vitamin C supplementation on exercise performance outcomes.

Study	Participants	Exercise	Timing	Supplementation	Result
Gomez-Cabrera et al. 2008 [11]	Human (15 men) and male Wistar rats	For humans: Static bicycle on 3 days/week for 8 weeks; for animals 5 days/week on an animal treadmill	For 8 weeks	For humans: 1 g/day vitamin C and for rats 0.24 mg/cm^2^ body surface area	Vitamin C reduced factors are PGC-1, nuclear respiratory factor 1, and mitochondrial transcription factor A. The exercise-induced expression of cytochrome C, SD, and glutathione peroxidase were blocked by vitamin C.
Roberts et al. 2011 [47]	Recreationally active males (n = 15)	high-intensity interval running protocol, 4 times per week	For 4 weeks	1 g/day	Training-induced improvements of VO_2max,_ 10 km time trial, and running economy were not affected by vitamin C.
Evans et al. 2017 [87]	Nine persons naive to resistance exercise (RE)	One RE bout was performed pre-supplementation and one was performed post-supplementation; RE bouts consisted of a warmup set of bodyweight pushups, three working sets (WS) of 10 isokinetic contracting push-pull repetitions, and one maximal effort set (ME) of five isokinetic contracting push-pull repetitions	For 28 days	250 mg every 12 h	Vitamin C supplementation increased peak muscular pushing force (PMF) and reduced exercise-induced oxidative stress.
Paschalis et al. 2016 [89]	recreationally trained healthy males (n = 20)	aerobic exercise to exhaustion	For 30 days	three vitamin C tablets/day (each tablet contained 333 mg of vitamin C)	The low vitamin C group had lower VO_2_ max values than the high vitamin C group. Vitamin C supplementation in this group marginally increased VO_2_ max.
Thompson et al. 2001 [90]	Active men (n = 16)	Aerobic: A prolonged intermittent shuttle-running test for 90 min	For 14 days	400 mg/day	Vitamin C supplementation had modest beneficial effects on muscle soreness and muscle function
Bryer and Goldfarb 2006 [91]	healthy men (n = 18)	Seventy eccentric elbow extensions with their non-dominant arm	for 2 weeks prior and 4 days after eccentric exercise	3 g/day	Vitamin C reduced muscle soreness and delayed CK increase. Muscle force and range of motion were not affected.
close et al. 2006 [92]	Physically active male subjects (n = 20)	Aerobic: Downhill running for 30 min at 60% VO_2_ max	For 2 h pre-, and for 14 days after exercise	1 g/day	Vitamin C attenuated RONS production following downhill running. No effect on DOMS was seen.
Connolly et al. 2006 [93]	24 subjects (male and female)	Anaerobic: 40 (2 × 20) maximal eccentric contractions of the elbow flexors	For 8 days (3 days prior to an exercise bouts and 5 days after)	3 g/day	Vitamin C had no effect on muscle soreness and muscle strength
Scalzo et al. 2018 [94]	Adults with T2D (n = 31) and healthy adults (n = 21)	Peak oxygen uptake was determined via graded exercise to exhaustion	Single dose	IV infusion of vitamin C (7.5 g)	Acute vitamin C infusion did not change VO_2_ peak
